# Characteristics of locoregional extension of unilateral nasopharyngeal carcinoma and suggestions for clinical target volume delineation

**DOI:** 10.1186/s13014-022-02017-2

**Published:** 2022-03-12

**Authors:** Zheng Wu, Lin Zhang, Qian He, Feiping Li, Hongzhi Ma, Yujuan Zhou, Hui Wang, Yaqian Han

**Affiliations:** 1 Department of Radiation Oncology, Hunan Cancer Hospital & the Affiliated Cancer Hospital of Xiangya School of Medicine, Central South University, Changsha, 410013 Hunan People’s Republic of China; 2 Department of Imaging, Hunan Cancer Hospital & the Affiliated Cancer Hospital of Xiangya School of Medicine, Central South University, Changsha, 410013 Hunan People’s Republic of China

**Keywords:** Nasopharyngeal carcinoma, Magnetic resonance imaging, Local invasion, Lymph node spread, Clinical target volume

## Abstract

**Background:**

To summarize the characteristics of local invasion and distribution of metastatic lymph nodes in unilateral nasopharyngeal carcinoma (NPC) by magnetic resonance imaging (MRI) to provide references for the optimization of clinical target volume.

**Methods:**

MRI and clinical data of 176 cases of unilateral NPC admitted to the Hunan Cancer Hospital from January 2019 to December 2019 were collected. Unilateral NPC was defined as a lesion confined to the one side of the nasopharynx and had not exceeded the midline as judged by MRI.

**Results:**

Ipsilateral levator veli muscle (63.1%, 111/176), tensor veli palatini muscle (55.7%, 98/176), parapharyngeal space (50.0%, 88/176), and prevertebral muscle (43.7%, 77/176) were more likely to be invaded. Contralateral parapharyngeal space and skull base foramina were not invaded. All local invasions presented as continuous invasion from gross lesions and discontinuous invasions were not observed. The overall lymph node metastatic rate was 89.8% (158/176), of which bilateral metastasis accounted for 56.3% (89/158), and ipsilateral metastasis accounted for 88.1% (155/176), which was higher than the contralateral metastatic rate (55.4%, 94/176) (*P* < 0.001). The most common regions of lymph node metastasis were level IIb (82.4%), VIIa (69.9%), IIa (54.0%), and III (54.0%). Only one patient had skipping lymph node metastasis (0.6%).

**Conclusion:**

Local invasion of unilateral NPC was characterized by continuous invasion from proximal to distal sites, and lymph node metastasis occurred from the upper to lower neck. Contralateral parapharyngeal space and skull base foramina had a very low probability of invasion, and routine prophylactic radiation may not be necessary.

## Background

Nasopharyngeal carcinoma (NPC) is a malignant tumor derived from nasopharyngeal epithelial cells. It is more common in South China and Southeast Asia, and radiotherapy is the most important radical cure for NPC. With the application of intensity-modulated radiotherapy, the prognosis of NPC has been significantly improved. The 5-year locoregional recurrence-free survival has reached 85–90% [1–3]. The incidence and severity of radiotherapy-induced xerostomia has also decreased significantly. However, patients who have been cured still have some sequelae of radiotherapy, such as hearing loss, temporal lobe injury, and endocrine dysfunction, which seriously affect quality of life [4, 5]. Therefore, further reducing the toxic and late side effects of radiotherapy is a key issue in NPC treatment.

In recent years, researchers have optimized the clinical target volume (CTV) of radiotherapy for NPC to reduce the side effects [6, 7]. Sanford et al. used individualized target delineation for NPC radiotherapy based on the tumor invasion trend [6]. It was not required to include all traditional high risk structures of NPC. For unilateral NPC, the contralateral parapharyngeal space and skull base foramina could not be included. Long-term follow-up showed that the overall 5-year local control rate reached 96%, and there was no recurrence of contralateral tissue structures. According to the current CTV delineation guidelines, parapharyngeal space and skull base foramina on both sides belong to high-risk regions, and require prophylactic radiation regardless of the location and stage of the tumor [8, 9]. However, as the primary lesion of unilateral NPC is relatively far from the contralateral parapharyngeal space and skull base foramina, and skull base fascia can act as a barrier, it is worth discussing the necessity of prophylactic radiation of contralateral tissues.

Unilateral NPCs constitute about 10% of all NPC cases [10, 11]. At present, data are limited regarding the characteristics of locoregional extension of this subtype of NPC. In the present study, we analyzed the characteristics of local invasion, as well as the distribution of lymph node metastasis in unilateral NPCs, and explored the feasibility of omitting prophylactic radiation of contralateral parapharyngeal space and the skull base foramina, and provided references for CTV optimization for unilateral NPCs.

## Materials and methods

### Patients

**Fig. 1:**
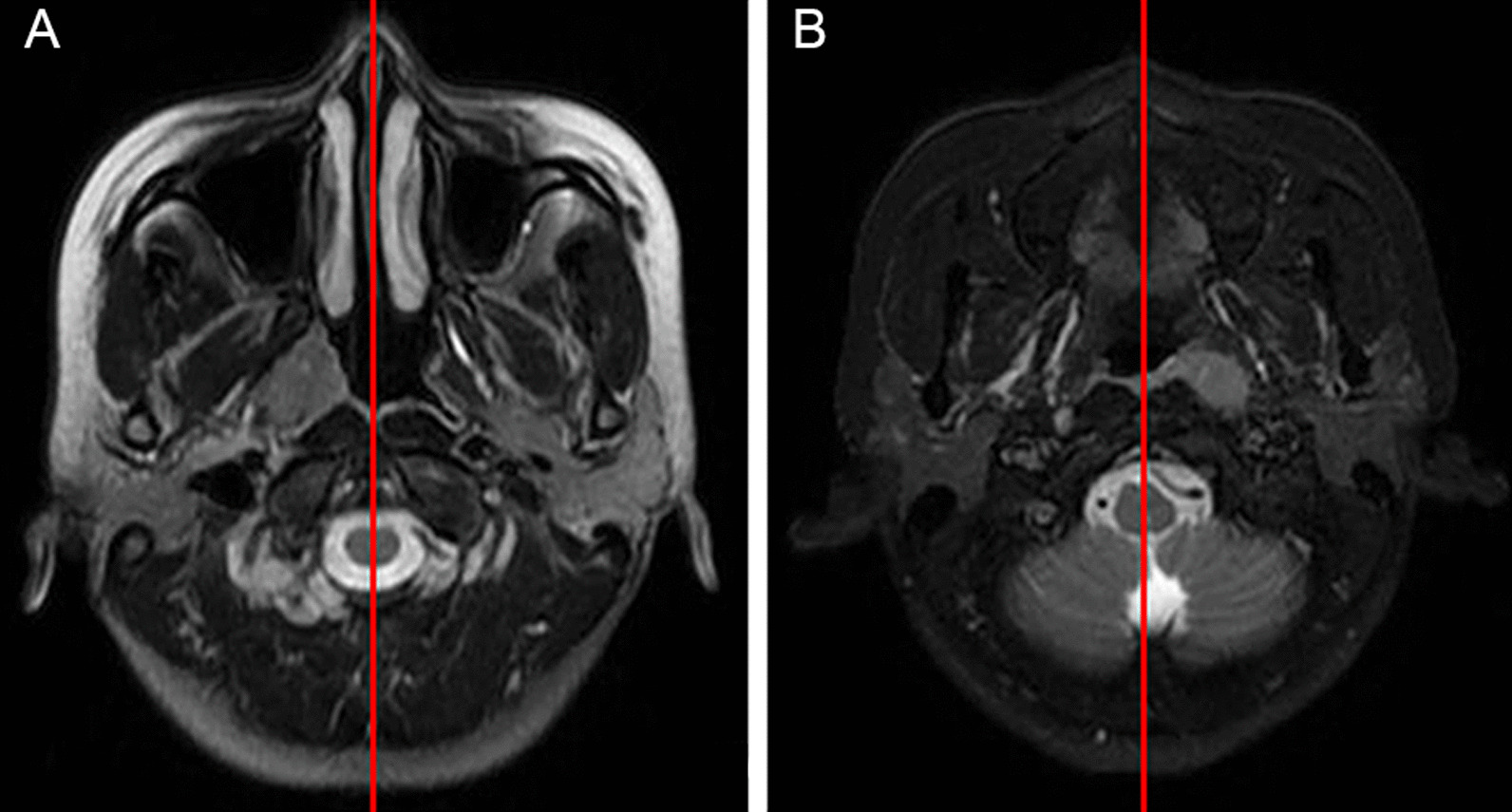
Criteria for diagnosis of unilateral nasopharyngeal carcinoma: **A** MRI of a lesion that did not exceed the midline of the nasopharynx; **B** MRI of a lesion that reached the midline but did not exceed the midline

Patients who were newly diagnosed with unilateral NPC and treated in the Department of Radiation Oncology of Hunan Cancer Hospital from January 2019 to December 2019 were enrolled. Unilateral NPC was defined as a lesion that was confined to the one side of the nasopharynx and had not exceeded the midline as determined by magnetic resonance imaging (MRI) (Fig. 1). Inclusion criteria were as follows: pathologically diagnosed as nasopharyngeal carcinoma; pretreatment nasopharynx and neck MRI imaging; pretreatment nasopharyngoscopy data; meeting the definition of unilateral NPC; and not received prior anti-tumor treatment. Patients with poor MRI imaging or undergoing nasopharyngeal mass resection or neck lymph node mass resection were excluded. All patients were staged using the 8th edition UICC/AJCC NPC staging system [12].

### MRI scan

All patients received an MRI scan of the nasopharynx and neck with a scan range from the middle of the temporal lobe to the thoracic entrance. The scanning plane was parallel to the third cervical vertebra in the coronal position, and perpendicular to the third cervical vertebra in the axial position. Slice thickness was 5 mm and slice gab was 0.5 mm. The scanning sequence included axial T1WI, T2WI, and diffusion-weighted imaging. After injection of the contrast agent gadopentetate meglumine (Gd-DTPA), the axial, coronal, and sagittal T1WI fat suppression sequence scans were performed.

### Diagnostic criteria

MRI images were reviewed by a senior imaging physician and a senior radiation oncologist independently, and disagreements were resolved by discussion. Tumor invasion was defined as low signal in T1WI, high signal in T2WI in MRI plain scan, and uneven enhancement in contrast scan [13]. Extra-nasopharynx cavity invasion was defined as invasion beyond the fascia of the pharyngeal skull base. Bone invasion was defined as disappearance of the high signal of fat in T1WI in the skull base bone, and obvious enhancement in contrast scan. Criteria of lymph node metastasis [14, 15]: ≥ 10 mm minimum diameter of lymph node in the largest cross-sectional image; ≥ 3 lymph nodes in the same high-risk region, and at least one lymph node having ≥ 8 mm minimum diameter in the largest cross-sectional image; ≥ 5 mm minimum diameter of posterior pharyngeal lymph nodes in the largest cross-sectional image; central necrosis or edge ring enhancement in any lymph nodes; extracapsular invasion of lymph nodes; significant shrinkage or disappear of lymph nodes in MRI image after chemotherapy or radiotherapy. Lymph node drainage levels were defined as previously described [16].

### Statistic analysis

Statistical analysis was performed using Statistical Package for the Social Sciences (SPSS) 22.0 (Chicago, IL, USA). The chi­square test was used to examine differences between categorical variables, and two­tailed values < 0.05 were considered significant.

## Results

### Patient characteristics

A total of 176 patients who met the definition of unilateral NPC and the enrollment criteria were included in the study. There were 124 males and 52 females with median age of 51.5 years old (24–74-years-old). There were 174 cases of non-keratinizing carcinoma and two cases of keratinizing squamous cell carcinoma. There were 82 cases with left-side lesions and 94 cases with right-side lesions. Patients’ characteristics are shown in Table 1.

**Table 1:** Patient characteristics

Characteristic	No. of patients (%)
*Sex*	
Male	124 (70.5)
Female	52 (29.5)
Median age (years old)	51.5 (24–74)
*Pathology*	
Non-keratinized carcinoma	174 (98.9)
Keratinized carcinoma	2 (1.1)
*Site*	
Right	94 (53.4)
Left	82 (46.6)
*T classification*	
T1	53 (30.1)
T2	50 (28.4)
T3	54 (30.7)
T4	19 (10.8)
*N classification*	
N0	18 (10.3)
N1	66 (37.5)
N2	52 (29.5)
N3	40 (22.7)
*M*	
M0	173 (98.3)
M1	3 (1.7)
*Clinic stage*	
I	9 (5.1)
II	46 (26.1)
III	67 (38.1)
IVa	51 (29.0)
IVb	3 (1.7)

### Characteristics of local invasion

In this cohort, tissue structures adjacent to the nasopharynx were most often invaded, including the ipsilateral levator veli muscle (63.1%, 111/176), tensor veli palatini muscle (55.7%, 98/176), parapharyngeal space (50.0%, 88/176), and prevertebral muscle (43.7%, 77/176). The orbit, hypopharynx, frontal sinus, contralateral parapharyngeal space, and skull base foramina were not invaded. Invasions showed continuity in all patients and discontinuous invasions were not observed. The frequency of invasion of various structures around the nasopharynx is shown in Table 2.

**Table 2:** Incidence of tumor invasion into anatomic sites surrounding nasopharynx

Anatomic site	No. of patients (%)	Anatomic site	No. of patients (%)
Levator veli muscle	111 (63.1)	Cavernous sinus	11 (6.3)
Tensor veli palatini muscle	98 (55.7)	Meninges	10 (5.7)
Parapharyngeal space	88 (50.0)	Nasal cavity	10 (5.7)
Prevertebral muscle	77 (43.7)	Sphenoidal sinus	9 (5.1)
Foramen lacerum	55 (31.2)	Inferior orbital fissure	8 (4.5)
Petrous apex	51 (29.0)	Parotid gland	5 (2.8)
Basis of sphenoid bone	43 (24.4)	Hypoglossal canal	3 (1.7)
Clivus	45 (25.6)	Maxillary sinus	2 (1.1)
Pterygoid process	34 (19.3)	Infratemporal fossa	2 (1.1)
Medial pterygoid muscle	32 (18.2)	Orbital apex	2 (1.1)
Pterygopalatine fossa	20 (11.4)	Jugular foramen	1 (0.6)
Foramen ovale	18 (10.2)	Cervical vertebrae	1 (0.6)
Oropharynx	16 (9.1)	Ethmoid sinus	1 (0.6)
Lateral pterygoid muscle	15 (8.5)		

### Distribution of metastatic lymph nodes

The overall lymph node metastatic rate was 89.8% (158/176), of which bilateral metastases accounted for 56.3% (89/158). The levels with the highest metastatic rate were IIb (82.4%), VIIa (69.9%), IIa (54.0%), and III (54.0%). Lymph node metastasis was not observed with levels Ia, IX, VI, and Xa. The ipsilateral lymph node metastatic rate (88.1%, 155/176) was significantly higher than contralateral metastatic rate (53.4%, 94/176) (χ^2^ = 12.937, *P* < 0.001). The distribution of metastatic lymph nodes is shown in Table 3.

**Table 3:** Distribution of metastatic lymph nodes

Level	Ipsilateral lymph node metastasis only	Contralateral lymph node metastasis only	Bilateral lymph node metastasis	Total
	No. of patients (%)	No. of patients (%)	No. of patients (%)	No. of patients (%)
Ia	0 (0)	0 (0)	0 (0)	0 (0)
Ib	8 (4.5)	0 (0)	3 (1.7)	11 (6.2)
IIa	56 (31.8)	9 (5.1)	30 (17.0)	95 (54.0)
IIb	70 (39.8)	7 (4.0)	68 (38.6)	145 (82.4)
III	68 (38.6)	5 (2.8)	22 (12.5)	95 (54.0)
IVa	29 (16.5)	3 (1.7)	3 (1.7)	35 (19.9)
IVb	8 (4.5)	2 (1.1)	0 (0)	10 (5.7)
Va	44 (25.0)	6 (3.4)	6 (3.4)	56 (31.8)
Vb	17 (9.7)	2 (1.1)	1 (0.6)	20 (11.4)
Vc	2 (1.1)	0 (0)	1 (0.6)	3 (1.7)
VI	0 (0)	0 (0)	0 (0)	0 (0)
VIIa	82 (46.6)	6 (3.4)	35 (19.9)	123 (69.9)
VIIb	3 (1.7)	0 (0)	0 (0)	3 (1.7)
VIII	4 (2.3)	0 (0)	0 (0)	4 (2.3)
IX	0 (0)	0 (0)	0 (0)	0 (0)
Xa	0 (0)	0 (0)	0 (0)	0 (0)
Xb	1 (0.6)	0 (0)	0 (0)	1 (0.6)

### Skipping lymph node metastasis and extra-regional lymph node metastasis

Only one patient had III and IVa metastases without ipsilateral IIa, IIb, and VIIa metastases, which was considered as skipping metastasis (0.6%, 1/176). All of the patients with IB, XIII, and Xb metastases had extensive ipsilateral lymph node metastases (more than three drainage levels were metastasized or the diameter of metastatic lymph nodes in the adjacent area was greater than 3 cm).

### Relationship between local invasion and lymph node metastasis

In the cohort, the lymph node metastatic rate was 83.0% (44/53) in T1-stage patients, 88.0% (44/50) in T2-stage patients, 96.3% (52/54) in T3-stage patients, and 94.7% (18/19) in T4-stage patients. There was no significant difference in lymph node metastatic rate among various T stages (χ^2^ = 6.249, *P* = 0.100).

There were 126 cases whose lesions were close to (≤ 0.5 cm) or arrived at the midline. The contralateral cervical lymph node metastatic rate was 56.3% (71/126) in these patients. Lesions in another 50 cases exceeded the 0.5 cm from the midline, and contralateral cervical lymph node metastatic rate was 46.0% (23/50) in these patients, which was not significantly different from the former (χ^2^ = 1.153, *P* = 0.283). In addition, there was no significant difference in the extent of local invasion between gross tumors with a distance of more than 0.5 cm and gross tumors with a distance of less than 0.5 cm from the midline (*P* > 0.05, all).

## Discussion

To our knowledge, this study is the first to focus on the characteristics of local invasion and lymph node metastasis of unilateral NPC. Our results showed that unilateral NPC tended to invade ipsilateral adjacent tissues. Ipsilateral muscles, parapharyngeal fat space, and skull base bones had a high chance of invasion. Ipsilateral intracranial structures such as meninges and cavernous sinuses could also be involved; this may occur through skull base foramina including ipsilateral foramen ovale cranium and foramen lacerum, or may be secondary to the invasion of surrounding tissues. Tissues far from primary lesions, e.g., hypopharynx, frontal sinus, and orbit, had only a low chance of invasion. Local invasions were all continuous in our cohort and discontinuous invasions were not observed. In summary, the local invasion in unilateral NPC showed characteristics of progressive involvement from proximal to distal sites.

The nasopharynx is the middle structure; thus, bilateral tissues are likely to be invaded. Previous studies have used MRI to study the characteristics of local invasion of NPC, which was found to follow certain pathways [13, 17, 18]. The surrounding structures have been classified as high, intermediate, or low risk to guide CTV delineation. In these studies, it was recommended that high risk structures on both sides be given prophylactic radiation [13, 18]. However, the studies also found that the probability of invasion of both sides was less than 10%, and the majority of NPCs showed an eccentric distribution. Furthermore, these studies did not compare local invasion of ipsilateral and contralateral tissues, especially for unilateral NPC. Our study indicated that the adjacent ipsilateral parapharyngeal space and skull base foramina were more likely to be affected, which is consistent with previous studies [13, 17, 18]. However, contralateral parapharyngeal space and other contralateral structures were not invaded, and skipping local invasion was also not observed. In light of this, we suggest equal prophylactic radiation on both side structures is not necessary for CTV delineation in unilateral NPC. Sanford et al. preliminarily reported the feasibility of omitting prophylactic radiation of the contralateral parapharyngeal space for unilateral NPC [6].

Another reason to include both sides of the parapharyngeal space and skull base foramina in the scope of prophylactic radiation was the concern about multicentric origin of NPC lesions. Mo et al. performed biopsies on contralateral nasopharyngeal tissues in 50 cases of unilateral NPC and found that the rate of contralateral pharyngeal crypt reached 18% [19]. However, it should be noted that they used plain CT and an electronic nasopharyngoscopy to diagnose unilateral NPC. Li et al. performed biopsies of the posterior wall of the contralateral pharyngeal roof as well as the contralateral pharyngeal crypt in 20 unilateral NPC cases judged by MRI [11]. Their result showed that five patients had subclinical lesions on the posterior wall of the contralateral pharyngeal roof and pharyngeal crypt biopsies were also positive for two patients. However, the primary lesions in four of the above five patients had invaded to the midline of the posterior wall. The researchers thereby ascribed the contralateral positive lesions to continuous subclinical infiltration of primary lesions instead of multicentric lesions, and suggested for patients with unilateral NPC but without contralateral LN metastasis or positive EBV-DNA level, CTV expansion to the contralateral normal mucosa of the nasopharynx probably could be reduced.

There is a rich lymphatic vascular network in nasopharynx mucosal tissue and lymphatic drainage on both sides of the neck. Previous studies have rarely discussed the characteristics of lymph node metastasis in unilateral NPC. Sun et al. summarized the distribution of metastatic lymph nodes in 112 patients with unilateral NPC, and found that the metastatic rate of contralateral cervical lymph nodes was only 8% [10]. Our study showed that the overall metastatic rate of lymph nodes was 89.8%. Although ipsilateral metastasis was more common (88.1%), contralateral metastasis was also high (53.4%). In addition, even with primary lesions not close to the midline, the contralateral lymph node metastatic rate was still 46.0%. Lymph node metastasis was not significantly associated with T stage, suggesting lymph node metastasis can occur in the early stage of NPC. Therefore, for unilateral NPC, early prophylactic radiation of both sides of the neck is still necessary.

Our results regarding the distribution of metastatic lymph nodes in lymphatic drainage regions were consistent with previous findings [18, 20, 21]. Levels IIa, IIb, and VIIa, which are considered the first station nodes, more commonly showed metastasis (metastatic rate exceeded 50%), followed by levels III, V, and IV, which had a > 20% metastatic rate. Extra-regional lymph nodes such as levels VI, VIII, IX, and X had the lowest metastatic rate (< 5%). Skipping lymph node metastasis happened in only one patient (0.6%), and metastases in other patients followed the rule of from the upper neck to the lower neck and station by station. All patients with metastases of extra-regional lymph nodes also had extensive ipsilateral cervical lymph node metastases, possibly due to obstruction of the lymphatic ducts and retrograde lymphatic drainage.

Our study suggested, for prophylactic radiation in unilateral NPC, the ipsilateral parapharyngeal space and skull base foramina were still high risk regions and should be included in CTV. The contralateral parapharyngeal space and skull base foramina were low risk regions, and routine prophylactic radiation may not be necessary. For prophylactic radiation of lymphatic drainage regions, the radiation scope and indications are consistent with the current guidelines [9, 22]. For N0 patients, the bilateral upper neck lymph nodes (levels II, III, Va, and VII) required prophylactic radiation; for N + patients, both bilateral upper and lower neck lymph nodes (levels II, III, IV, V, and VII) required prophylactic radiation. For extra-regional lymph nodes, routine prophylactic radiation was not indispensable; however, if there was extensive ipsilateral lymph node metastasis, it was necessary to pay close attention to extra-regional lymph nodes. A large-scale clinical study is needed to determine whether the contralateral parapharyngeal space and skull base foramina can be omitted from prophylactic radiation.

Our study had some limitations. Although MRI was the first choice for judging the extent of NPC invasion, false positive or false negative results were possible. Because contralateral nasopharyngeal mucosa biopsies were not performed, we were unable to adequately judge invasion of the contralateral nasopharyngeal mucosa. Therefore, we also reviewed the electronic nasopharyngoscope data of all patients to ensure accuracy of the diagnosis. In addition, the sample size of this study was relatively small, and the results may be biased. We will collect more data to verify our findings.

## Conclusions

Local invasion of unilateral NPC was characterized by continuous spread from proximal to distal sites. Adjacent ipsilateral tissues were invaded more easily, and the contralateral parapharyngeal space and skull base foramina were at low risk of invasion. Lymph node metastasis followed the rule of from the upper neck to the lower neck, and bilateral cervical lymph node metastasis was common. In the setting of CTV for unilateral NPC, the ipsilateral parapharyngeal space and cranial foramina still needed to be included in the scope of prophylactic radiation, while routine prophylactic radiation of contralateral structures such as the parapharyngeal space may not be necessary. Even for unilateral NPC, both sides of the neck required prophylactic radiation.

## Data Availability

All data generated or analyzed during this study are included in this published article.

## Abbreviations

NPC: Nasopharyngeal carcinoma
CTV: Clinical target volume
MRI: Magnetic resonance imaging
